# Effectiveness of Ocrelizumab on Disease Progression and Disability Status in Multiple Sclerosis Patients: A Two-Year Prospective Cohort Study

**DOI:** 10.3390/jcm14020553

**Published:** 2025-01-16

**Authors:** Amanda Claudia Schuldesz, Raluca Tudor, Amalia Cornea, Dorina Nicola Geni, Irina Nicoleta Lata, Mihaela Simu

**Affiliations:** 1 Doctoral School, “Victor Babes” University of Medicine and Pharmacy Timisoara, 300041 Timisoara, Romania; amanda.schuldesz@umft.ro (A.C.S.); nicola.geni@umft.ro (D.N.G.); irina.dragota@umft.ro (I.N.L.); 2 Discipline of Neurology, “Victor Babes” University of Medicine and Pharmacy Timisoara, 300041 Timisoara, Romania; amalia.cornea@umft.ro (A.C.); simu.mihaela@umft.ro (M.S.)

**Keywords:** multiple sclerosis, ocrelizumab, disease progression, disability status, MRI

## Abstract

**Background and Objectives:** Multiple sclerosis (MS) is a chronic autoimmune disorder characterized by inflammation and neurodegeneration. Ocrelizumab, a humanized anti-CD20 monoclonal antibody, has shown promise in reducing disease activity in MS patients. This prospective study aims to assess the effectiveness of ocrelizumab in reducing confirmed disability progression in patients with relapsing-remitting MS (RRMS) and secondary progressive MS (SPMS) over a two-year period. By evaluating clinical data, and MRI findings, this study seeks to provide comprehensive insights into ocrelizumab’s impact on disease dynamics and disability. **Materials and Methods:** Ninety-eight patients aged 18 to 65 with confirmed MS were enrolled under ocrelizumab therapy at the Neurology Department of “Pius Brinzeu” Clinical Emergency Hospital in Romania between July 2020 and July 2024. Participants were assessed at baseline and every six months over two years. The key outcomes measured were changes in the Expanded Disability Status Scale (EDSS) as a measure of confirmed disability progression (CDP), annualized relapse rate (ARR), and MRI findings. **Results:** Over the two-year period, the mean EDSS score significantly decreased from 5.2 ± 1.8 to 4.6 ± 1.7 (mean change = −0.6 ± 0.9; *p* = 0.032), indicating improved neurological function. The proportion of patients experiencing relapses dropped markedly from 61.2% to 14.3% (*p* < 0.001). The MRI results showed significant reductions in patients with new or enlarging T2 lesions from 68.4% to 27.6% (*p* < 0.001) and gadolinium-enhancing lesions from 44.9% to 15.3% (*p* < 0.001). Patients previously treated with natalizumab exhibited a greater reduction in EDSS scores (−1.0 ± 0.8; *p* = 0.001) compared to other treatments. Multivariate regression identified the baseline EDSS score (β = 0.65; *p* < 0.001), previous natalizumab use (β = −0.30; *p* = 0.013), and age at diagnosis (β = 0.02; *p* = 0.048) as significant predictors of two-year EDSS scores. While markers of active inflammation decreased, the proportion of patients with brain atrophy increased from 31.6% to 43.9% (not statistically significant; *p* = 0.105). SPMS patients had higher rates of brain atrophy at baseline (61.1% vs. 25.0%; *p* = 0.007) and at two years (100.0% vs. 31.3%; *p* < 0.001) compared to RRMS patients. **Conclusions:** Ocrelizumab effectively reduced disease activity and improved neurological disability over two years in both RRMS and SPMS patients. Significant reductions in relapse rates and MRI markers of inflammation were observed. Previous natalizumab treatment was associated with greater improvements. Despite these benefits, the progression of neurodegeneration, particularly brain atrophy in SPMS patients, underscores the need for additional strategies targeting neurodegenerative aspects of MS.

## 1. Introduction

Multiple sclerosis (MS) is a chronic inflammatory disease of the central nervous system characterized by demyelination and neurodegeneration, leading to neurological disability and impaired quality of life [1]. The heterogeneity of MS manifestations poses challenges in disease management and underscores the need for effective therapeutic strategies [2].

Disease-modifying therapies (DMTs) have transformed the MS treatment landscape by reducing relapse rates and slowing disease progression [3]. Among these, ocrelizumab, a humanized monoclonal antibody targeting CD20-positive B cells, has shown efficacy in both relapsing and primary progressive forms of MS [4]. Clinical trials have demonstrated ocrelizumab’s ability to reduce relapse rates and delay disability progression [5].

Despite clinical trial success, real-world data on ocrelizumab’s long-term effectiveness and impact on quality of life remain limited [6]. Prospective studies are essential to understand the drug’s performance outside controlled environments and across diverse patient populations [7]. Such studies can provide insights into treatment adherence, safety profiles, and patient-reported outcomes, which are crucial for comprehensive MS management [8].

Quality of life in MS patients is affected by physical disability, cognitive impairment, and psychological factors [9]. Previous research indicates that improvements in clinical measures do not always translate into enhanced quality of life [10]. Therefore, evaluating patient-reported outcomes alongside clinical and radiological assessments is vital [11]. Moreover, magnetic resonance imaging (MRI) serves as a key tool in monitoring MS disease activity and treatment response [12]. MRI findings, such as T2 lesion load and gadolinium-enhancing lesions, correlate with clinical outcomes and can guide therapeutic decisions [13]. 

In the pivotal OPERA I and OPERA II trials, ocrelizumab was shown to reduce the annualized relapse rate by approximately 46% compared to interferon beta-1a and significantly decreased the number of new or enlarging T2 lesions and gadolinium-enhancing lesions on MRI scans [4,5]. Additionally, the ORATORIO trial established ocrelizumab’s effectiveness in primary progressive MS, showing a marked reduction in disability progression as measured by the Expanded Disability Status Scale (EDSS) over a two-year period [4]. Real-world studies further corroborate these findings, indicating that ocrelizumab not only stabilizes but can also improve disability status and minimize disease activity across diverse patient populations [14].

This prospective study aims to evaluate the effectiveness of ocrelizumab in reducing DP and improving the disability status in both RRMS and PPMS patients over a two-year period. By analyzing clinical data, and MRI findings, we seek to provide real-world evidence of ocrelizumab’s impact. Understanding the multifaceted effects of ocrelizumab can inform clinical practice and optimize patient care. This study also explores subgroup analyses to identify factors associated with better treatment response, which could aid in personalizing MS therapy [15].

## 2. Materials and Methods

### 2.1. Study Design and Setting

This prospective, observational cohort study was conducted at the Neurology Department of “Pius Brinzeu” Clinical Emergency Hospital in Western Romania between July 2020 and July 2024. The study aimed to evaluate the effectiveness of ocrelizumab in patients with multiple sclerosis over a two-year follow-up period. Ethical approval was obtained from the Institutional Review Board (IRB) of the hospital, and written informed consent was obtained from all participants prior to enrollment. The study was conducted in accordance with the Declaration of Helsinki and adhered to Good Clinical Practice guidelines.

### 2.2. Participants and Inclusion Criteria

Patients eligible for inclusion were adults aged 18 to 65 years with a confirmed diagnosis of relapsing-remitting MS (RRMS) or secondary progressive MS (SPMS) according to the 2017 revised McDonald criteria [16]. Inclusion criteria required that patients were treatment-naïve or had an inadequate response to at least one previous disease-modifying therapy (DMT). All patients were scheduled to initiate ocrelizumab therapy as part of their clinical care.

Exclusion criteria included primary progressive MS (PPMS), previous treatment with ocrelizumab, pregnancy or breastfeeding, active infection, history of malignancy, or any other significant medical condition that could interfere with the study. Patients with contraindications to MRI or those unable to complete the required assessments were also excluded. Patients who discontinued ocrelizumab treatment were also excluded from the final analysis.

A total of 110 patients were initially enrolled in the study. During the two-year follow-up period, 12 patients discontinued ocrelizumab treatment. The reasons for discontinuation included adverse events (n = 5), lack of efficacy (n = 4), and personal reasons (n = 3). These patients were excluded from the final analysis, resulting in 98 patients who completed the study. Baseline demographic and clinical characteristics, including age, gender, disease duration, MS subtype, and prior treatments, were recorded. Patients were stratified into subgroups based on gender, age at diagnosis (≤25 years vs. >25 years), and previous treatments for subgroup analyses.

### 2.3. Data Collection and Outcome Measures

Data collection for the study was comprehensive, encompassing clinical evaluations, laboratory tests, and MRI scans, all conducted at baseline and then at six-month intervals throughout the two-year follow-up period. Clinical assessments included the use of the Expanded Disability Status Scale (EDSS) by trained neurologists who were blinded to subgroup assignments, to measure neurological impairment and disability [17]. Additionally, relapse assessment was defined by the emergence of new or worsening neurological symptoms that lasted at least 24 h, occurring in the absence of fever or infection. Confirmed disability progression (CDP) was measured through the EDSS at 6 months, and was identified as an increase of ≥1.5 points with baseline EDSS = 0; ≥1.0 with baseline EDSS score ≤5.0; and ≥0.5 points with baseline EDSS > 5.5. Moreover, the occurrence of relapses was expressed as annualized relapse rate (ARR), comparing the period before and after starting the DMT.

MRI assessments were standardized using a 1.5 Tesla scanner to capture T1-weighted, T2-weighted, and fluid-attenuated inversion recovery (FLAIR) images, both with and without gadolinium contrast enhancement. The key outcomes from these scans included the number of new or enlarging T2 lesions, the presence of gadolinium-enhancing lesions, and changes in lesion burden and brain atrophy. This structured approach allowed for a detailed tracking of disease progression through imaging.

To accurately assess changes in brain, corpus callosum, and spinal cord atrophy, this study employed GE’s AW VolumeShare 5 software for volumetric analysis. MRI sequences utilized included T1-weighted, T2-weighted, and FLAIR images for detailed visualization of black holes and spinal cord lesions. Regarding spinal cord lesions, the analysis specifically focused on confluent lesions due to their higher clinical relevance and stronger correlation with disability progression in MS patients.

Stratification was initially conducted by gender and age at MS diagnosis, with a cutoff of 25 years, to explore developmental and potentially hormonal influences on disease progression and therapy effectiveness. This age threshold was chosen based on previous studies suggesting potential differences in disease manifestation, confirming a bimodal peak, corresponding to the 25–34 and 45–54 age groups [18]. Subsequent stratifications involved previous disease-modifying therapy and MS subtype (RRMS vs. SPMS) to assess the impact of prior treatments and disease stage on ocrelizumab’s efficacy. Each stratification aimed to refine our analysis and provide insights tailored to specific patient groups, though not all stratifications were used in each analysis table.

### 2.4. Statistical Analysis

Statistical analyses were conducted using SPSS version 26.0 (IBM Corp., Armonk, NY, USA), where continuous variables were expressed as means ± standard deviations (SD) and categorical variables as frequencies and percentages. The distribution of the data was evaluated for normality using the Shapiro–Wilk test. Within-group comparisons of baseline and two-year follow-up data utilized paired *t*-tests for normally distributed continuous variables and Wilcoxon signed-rank tests for those not normally distributed. For between-group comparisons involving multiple groups, an analysis of variance (ANOVA) was employed for normally distributed data, while the Kruskal–Wallis test was used for non-normal data. Post hoc analyses with Bonferroni correction were conducted to control the family-wise error rate and adjust for multiple comparisons.

Categorical variables were analyzed using Chi-square tests or Fisher’s exact tests to compare proportions between groups. In terms of multivariate analyses, multiple linear and logistic regression models were used to identify factors independently associated with outcomes, including variables with a *p*-value of less than 0.10 in univariate analyses and interaction terms to assess potential effect modification by factors such as gender and previous treatments. Missing data were evaluated for patterns and handled using statistically appropriate methods, including multiple imputations when necessary.

Statistical significance was determined at a *p*-value of less than 0.05 for all analyses unless specified otherwise due to adjustments for multiple comparisons, and all statistical tests were conducted as two-tailed. This comprehensive analytical approach ensured rigorous evaluation and interpretation of the study’s findings. 

## 3. Results

Table 1 presents the baseline characteristics of the study population, stratified by gender and age at diagnosis. The total cohort comprised 98 patients, with 38 males and 60 females. The mean age was 44.2 years, with males slightly older than females, although the difference was not statistically significant (*p* = 0.215). When stratified by age at diagnosis, significant differences were observed; patients diagnosed at age ≤ 25 years were significantly younger (mean age 24.1 years) compared to those diagnosed after 25 years (mean age 49.3 years, *p* < 0.001).

Disease duration was slightly longer in patients diagnosed after 25 years compared to those diagnosed earlier (10.5 vs. 8.6 years, *p* = 0.046). Baseline EDSS scores did not differ significantly between genders or age groups. The majority of patients had relapsing-remitting MS (RRMS), with no significant differences in MS subtype distribution by gender or age group. Previous treatment exposure was similar across groups, as was the baseline lymphocyte count.

Table 2 summarizes the changes in clinical and MRI outcomes over the two-year follow-up period. The mean EDSS score decreased significantly from 5.2 at baseline to 4.6 at two years (mean change −0.6, *p* = 0.032), indicating an improvement in neurological disability. The proportion of patients with confirmed disability progression decreased dramatically from 61.2% to 14.3% (*p* < 0.001), highlighting ocrelizumab’s effectiveness in reducing disease activity. The MRI findings showed a significant reduction in disease activity, with the number of patients exhibiting new or enlarging T2 lesions decreasing from 68.4% to 27.6% (*p* < 0.001). Similarly, the presence of gadolinium-enhancing lesions decreased from 44.9% to 15.3% (*p* < 0.001). Additionally, lymphocyte counts decreased significantly over the two years (mean change −858 cells/mm^3^, *p* < 0.001), consistent with ocrelizumab’s mechanism of action targeting B cells. 

Table 3 presents the changes in EDSS scores over two years, stratified by previous treatment. Patients previously treated with natalizumab showed a significant mean reduction in EDSS score of −1.0 (*p* = 0.001), indicating substantial improvement in disability. Those who had received interferon beta-1a or glatiramer acetate showed smaller, non-significant reductions. Patients with no previous treatment also exhibited a slight, non-significant decrease in EDSS scores. The between-group comparison revealed a statistically significant difference (*p* = 0.008), suggesting that prior treatment with natalizumab may be associated with a greater response to ocrelizumab.

Table 4 shows the results of a multivariate linear regression analysis predicting the two-year EDSS score. The baseline EDSS score was the strongest predictor of the two-year EDSS score (beta coefficient = 0.65, *p* < 0.001), indicating that higher initial disability is associated with higher disability after two years. Previous use of natalizumab was significantly associated with a lower two-year EDSS score (beta = −0.30, *p* = 0.013), suggesting that patients previously treated with natalizumab may experience greater benefits from ocrelizumab therapy. Age at diagnosis was also a significant predictor (beta = 0.02, *p* = 0.048), with older age at diagnosis associated with higher EDSS scores. Gender and MS subtype approached statistical significance, with males and SPMS subtype trending towards higher EDSS scores, but these did not reach the conventional *p* < 0.05 threshold. Disease duration was not a significant predictor in this model. 

The proportion of patients with new or enlarging T2 lesions decreased significantly in both RRMS and SPMS groups over the two-year period. In RRMS patients, the proportion decreased from 68.8% at baseline to 25.0% at two years (*p* < 0.001). SPMS patients also showed a significant reduction from 66.7% to 38.9% (*p* = 0.003). Between-group comparisons did not show a statistically significant difference at either timepoint (Table 5). 

At baseline, 43.8% of RRMS patients and 50.0% of SPMS patients had gadolinium-enhancing lesions. After two years of ocrelizumab treatment, the proportion decreased significantly to 12.5% in RRMS patients (*p* < 0.001) and 27.8% in SPMS patients (*p* = 0.005). While both groups showed significant reductions, there was no statistically significant difference between RRMS and SPMS patients at either timepoint (Table 6). 

Over the two-year period, the MRI findings indicated that the proportion of patients with brain atrophy increased from 31.6% to 43.9% (*p* = 0.105) and corpus callosum atrophy rose from 21.4% to 34.7% (*p* = 0.056), though these changes were not statistically significant. Similarly, increases were observed in black holes (26.5% to 39.8%, *p* = 0.069), cervical confluent lesions (16.3% to 24.5%, *p* = 0.215), and cervical focal atrophy (11.2% to 19.4%, *p* = 0.165). In contrast, there were significant decreases in the number of patients with ≥3 new T2 lesions (from 40.8% to 15.3%, *p* < 0.001) and those with gadolinium-enhancing lesions (from 44.9% to 16.3%, *p* < 0.001), suggesting a reduction in active disease activity over time (Table 7). 

Findings over two years stratified by MS subtype showed that patients with secondary progressive MS (SPMS) had significantly higher rates of brain atrophy compared to those with relapsing-remitting MS (RRMS). At baseline, 61.1% of SPMS patients exhibited brain atrophy versus 25.0% of RRMS patients (*p* = 0.007), and by two years, all SPMS patients (100.0%) had brain atrophy compared to 31.3% of RRMS patients (*p* < 0.001). Similarly, significant differences were observed in corpus callosum atrophy and black holes at two years (*p* = 0.001 and *p* < 0.001, respectively), as presented in Table 8. 

Additional findings (Table 9) indicate that baseline brain atrophy was a significant predictor of disease progression over two years, with an odds ratio of 2.6 (95% CI: 1.25–5.40, *p* = 0.012). Higher baseline EDSS scores (OR = 1.35, *p* = 0.001) and older age at diagnosis (OR = 1.05, *p* = 0.018) were also significant predictors, while previous use of natalizumab was associated with reduced progression risk (OR = 0.58, *p* = 0.038); gender and MS subtype did not significantly predict progression. 

## 4. Discussion

### 4.1. Literature Findings

The findings from this prospective study highlight the significant impact of ocrelizumab on disease progression and disability status in multiple sclerosis patients over a two-year period. The reduction in EDSS scores indicates not only a halting of disease progression but also a potential reversal of neurological impairment. This aligns with ocrelizumab’s mechanism of action in depleting CD20-positive B cells, thereby reducing inflammatory activity within the central nervous system [14].

The MRI outcomes corroborated the clinical improvements, with substantial reductions in new or enlarging T2 lesions and gadolinium-enhancing lesions. These imaging findings suggest a robust response to ocrelizumab in stabilizing disease activity, which is critical in managing MS progression [19].

The results indicate that patients previously treated with natalizumab not only exhibited a greater reduction in EDSS scores but also showed a markedly lower rate of relapses compared to those who had not received natalizumab. Additionally, MRI analyses revealed that these patients had fewer new or enlarging T2 lesions and a reduced presence of gadolinium-enhancing lesions over the two-year period. These findings suggest that prior treatment with natalizumab may prime patients for a more favorable response to ocrelizumab, potentially due to a more controlled inflammatory environment at the initiation of ocrelizumab therapy [20].

Age at diagnosis emerged as a significant factor, with younger patients experiencing greater cognitive benefits [21,22]. This underscores the importance of early diagnosis and treatment initiation to optimize outcomes [23]. Gender differences were observed in the univariate analyses, with females showing slightly greater improvements, although these did not reach statistical significance in multivariate models. Further research may be needed to explore potential gender-specific responses to ocrelizumab.

Although, in our study, the QoL was only indirectly assessed through the EDSS as an improvement in disability scores, other studies focused on this aspect using standardized questionnaires for multiple sclerosis patients. For example, the study by Glanz et al. [24] reported significant improvements in both physical and mental health-related quality of life (HRQOL) domains after 12 months of ocrelizumab treatment in a cohort of 130 MS patients, utilizing the Medical Outcomes Study SF-36 and Neuro-QoL scales. Notably, there were improvements in SF-36 Role-Physical, General Health, Vitality, Role-Emotional, and Mental Health, as well as in Neuro-QoL domains such as Positive Affect and Fatigue. Similarly, the Russian study by Boyko et al. [25], which included 38 MS patients, showed considerable enhancements in HRQOL indices on the SF-36 and MS-specific MusiQoL questionnaires after 12 months of treatment, with reductions in depression severity evident after just 6 months. 

The study by Healy et al. [26] examined the associations between different intensities of physical activity (PA) and health-related quality of life in individuals with multiple sclerosis over a period of two years. Notably, while strenuous and moderate PA were weakly associated with many quality of life outcomes, mild PA showed only a weak correlation with lower extremity function and no significant associations at baseline when other PAs were absent. However, increases in mild PA over two years were moderately associated with improvements in upper extremity function and mental health components. In a similar manner, the study by Hersh et al. [27] focused on the comparison of time to clinically meaningful improvement in HRQOL in neurological disorders, assessing patients treated with natalizumab versus ocrelizumab. They found that natalizumab led to significantly shorter times to improvement in cognitive function, sleep disturbance, and social role participation and satisfaction. 

Another study by Healy et al. [28] explored the correlations between three patient-reported outcome measures in persons with multiple sclerosis, finding strong correlations between similar constructs across these tools. Specifically, the PROMIS-10 Global Physical Health score demonstrated a robust correlation with the SF-36 Physical Component Score (r = 0.798), and its Global Mental Health score was similarly linked with the SF-36 Mental Component Score (r = 0.726). However, correlations between PROMIS-10 and some NeuroQoL domains were weaker, suggesting that these measures might capture different aspects of health-related quality of life (HRQOL). In a similar manner, the PRO-MSACTIVE study by Manchon et al. [29] provided insights into the efficacy, safety, and patient-reported outcomes in patients with active relapsing MS treated with ocrelizumab. At 48 weeks, significant proportions of patients were free of disease activity, with 63.3% overall and differences noted between RRMS and SPMS. Patient-reported outcomes remained stable throughout the study, indicating a maintained or improved quality of life under treatment. 

Moreover, Barcutean et al. observed a significant reduction in relapse rates following the initiation of first-line disease-modifying therapies in a cohort of 523 Caucasian patients with relapsing-remitting MS [30]. Notably, the time to reach an EDSS of 3.0 and 6.0 varied significantly based on demographic factors such as onset topography and urban origin, although relapses did not significantly affect the time to reach these EDSS scores. In a similar manner, the study by Simone et al. [31] analyzed 3777 MS patients and revealed that age significantly influences the rate of disability progression, with pediatric-onset MS patients exhibiting a less steep increase in EDSS scores over time compared to adult-onset MS (AOMS) and late-onset MS.

The results of this study are consistent with previous clinical trials demonstrating ocrelizumab’s efficacy in reducing relapse rates and disability progression [5]. The significant improvements in MRI outcomes align with findings from the OPERA I and II trials [5]. Real-world studies have reported similar benefits, although long-term data remain limited [6]. This study adds to the literature by providing prospective evidence over a two-year period, with comprehensive assessments of clinical and radiological outcomes.

Our study demonstrates a significant reduction in EDSS scores and a marked improvement in MRI markers among patients treated with ocrelizumab, indicating reduced disease activity and improved neurological function. This contrasts with the findings from the study by Houtchens and Howard [32], where about one-third of highly disabled patients experienced disease stabilization, and a quarter reported disability worsening over time. While we observed a substantial decrease in relapse rates and new or enlarging T2 lesions, the other study noted that a significant proportion of patients continued treatment without side effects, yet almost half discontinued due to various reasons including a lack of benefit. These differing outcomes emphasize the potential for more pronounced therapeutic effects of ocrelizumab in patients with less severe disability at initiation compared to those with higher levels of disability.

The findings support the use of ocrelizumab as an effective therapeutic option in managing MS, with benefits extending beyond disease stabilization to improvements in disability status. Personalized treatment strategies considering patient age, previous treatments, and disease subtype may enhance outcomes. The early initiation of ocrelizumab in eligible patients could be advocated, especially for those diagnosed at a younger age, to maximize functional benefits. 

### 4.2. Study Limitations

This study has several limitations. Although prospective, the observational design may still be subject to biases inherent in non-randomized studies. The absence of a control group limits the ability to attribute improvements solely to ocrelizumab, as natural disease fluctuations cannot be entirely ruled out. The sample size, while adequate for primary analyses, may limit the power to detect smaller differences in subgroup analyses. The study was conducted at a single center, which may affect the generalizability of the findings to broader populations. Data on long-term safety and rare adverse events were not the focus of this study and require ongoing surveillance. Future studies with larger, multicenter cohorts and randomized controlled designs are needed to confirm these findings and explore the long-term efficacy and safety of ocrelizumab.

## 5. Conclusions

Ocrelizumab demonstrates significant efficacy in reducing disease progression and enhancing the disability status in MS patients over a two-year period. The improvements in clinical disability in relation to MRI changes highlight ocrelizumab’s role as a valuable therapeutic agent in MS management. Personalized treatment approaches considering patient demographics, such as age at diagnosis and previous treatments, may optimize outcomes. The early initiation of therapy, particularly in younger patients, appears beneficial. Further research is essential to explore long-term effects and establish comprehensive care strategies for MS patients, incorporating both pharmacological and supportive interventions. 

## Figures and Tables

**Table 1:** Baseline characteristics of patients stratified by gender and age at diagnosis.

Variable	Total (n = 98)	Male (n = 38)	Female (n = 60)	Age ≤ 25 (n = 35)	Age > 25 (n = 63)	*p*-Value (Gender)	*p*-Value (Age Group)
Age (years)	44.2 ± 9.5	45.6 ± 10.2	43.1 ± 8.9	24.1 ± 1.3	49.3 ± 6.8	0.215	<0.001
Disease Duration (years)	9.8 ± 4.7	10.2 ± 5.1	9.5 ± 4.4	8.6 ± 3.9	10.5 ± 5.0	0.528	0.046
Baseline EDSS Score	5.2 ± 1.8	5.4 ± 1.9	5.1 ± 1.7	4.8 ± 1.5	5.4 ± 1.9	0.462	0.089
MS Subtype (RRMS/SPMS)	80/18	31/7	49/11	30/5	50/13	0.801	0.933
Previous Treatment (Yes/No)	72/26	28/10	44/16	26/9	46/17	0.93	0.987
Lymphocyte Count (cells/mm^3^)	3472 ± 279	3490 ± 285	3459 ± 275	3480 ± 280	3469 ± 278	0.738	0.892

EDSS—Expanded Disability Status Scale; MS—Multiple Sclerosis.

**Table 2:** Changes in EDSS scores and MRI outcomes from baseline to two years.

Outcome	Baseline	Two Years	Mean Change	*p*-Value
EDSS Score	5.2 ± 1.8	4.6 ± 1.7	−0.6 ± 0.9	0.032
CDP	60 (61.2%)	14 (14.3%)	−46.90%	<0.001
New/Enlarging T2 Lesions	67 (68.4%)	27 (27.6%)	−40.80%	<0.001
Gd-Enhancing Lesions	44 (44.9%)	15 (15.3%)	−29.60%	<0.001
Lymphocyte Count	3472 ± 279 cells/mm^3^	2614 ± 287 cells/mm^3^	−858 ± 410 cells/mm^3^	<0.001
ARR	1.25 ± 0.47 relapses/year	0.63 ± 0.36 relapses/year	−0.62 ± 0.41 relapses/year	<0.001
NEDA	20 (20.4%)	57 (58.2%)	37.80%	<0.001

EDSS—Expanded Disability Status Scale; CDP—Confirmed Disability Progression; ARR—Annualized Relapse Rate; NEDA—No Evidence of Disease Activity.

**Table 3:** Changes in EDSS scores over two years stratified by previous treatment.

Previous Treatment	n	Baseline EDSS	Two-Year EDSS	Mean Change	*p*-Value
Natalizumab	25	5.0 ± 1.6	4.0 ± 1.4	−1.0 ± 0.8	0.001
Interferon Beta-1a	26	5.3 ± 1.7	4.9 ± 1.6	−0.4 ± 0.9	0.075
Glatiramer Acetate	21	5.4 ± 1.9	5.1 ± 1.8	−0.3 ± 0.7	0.102
No Previous Treatment	26	5.0 ± 1.8	4.8 ± 1.7	−0.2 ± 0.6	0.150
Between Groups					0.008

EDSS—Expanded Disability Status Scale.

**Table 4:** Multivariate linear regression analysis of factors predicting two-year EDSS score.

Variable	Beta Coefficient	Standard Error	*p*-Value
Baseline EDSS Score	0.65	0.1	<0.001
Gender (Male = 1, Female = 0)	−0.15	0.08	0.067
Age at Diagnosis	0.02	0.01	0.048
Previous Natalizumab Use	−0.3	0.12	0.013
Disease Duration	0.01	0.02	0.662
MS Subtype (SPMS = 1, RRMS = 0)	0.28	0.15	0.06

RRMS—Relapsing-Remitting Multiple Sclerosis; SPMS—Secondary Progressive Multiple Sclerosis; EDSS—Expanded Disability Status Scale; MS—Multiple Sclerosis.

**Table 5:** Changes in the number of new or enlarging T2 lesions over two years stratified by MS subtype.

Timepoint	RRMS (n = 80)	SPMS (n = 18)	*p*-Value
Baseline	55 (68.8%)	12 (66.7%)	0.841
Two Years	20 (25.0%)	7 (38.9%)	0.214
*p*-value	<0.001	0.003	

RRMS—Relapsing-Remitting Multiple Sclerosis; SPMS—Secondary Progressive Multiple Sclerosis.

**Table 6:** Changes in gadolinium-enhancing lesions over two years stratified by MS subtype.

Timepoint	RRMS (n = 80)	SPMS (n = 18)	*p*-Value
Baseline	35 (43.8%)	9 (50.0%)	0.62
Two Years	10 (12.5%)	5 (27.8%)	0.103
*p*-value	<0.001	0.005	

RRMS—Relapsing-Remitting Multiple Sclerosis; SPMS—Secondary Progressive Multiple Sclerosis.

**Table 7:** MRI findings and changes over two years.

MRI Variable	Baseline n (%)	Two Years n (%)	*p*-Value (Chi-Square)
Brain Atrophy	31 (31.6%)	43 (43.9%)	0.105
Corpus Callosum Atrophy	21 (21.4%)	34 (34.7%)	0.056
Black Holes	26 (26.5%)	39 (39.8%)	0.069
Cervical Confluent Lesions	16 (16.3%)	24 (24.5%)	0.215
Cervical Focal Atrophy	11 (11.2%)	19 (19.4%)	0.165
Number of Cervical Lesions ≥ 3	19 (19.4%)	12 (12.2%)	0.24
Thoracic Confluent Lesions	13 (13.3%)	17 (17.3%)	0.552
Thoracic Focal Atrophy	9 (9.2%)	15 (15.3%)	0.276
Patients with ≥3 New T2 Lesions	40 (40.8%)	15 (15.3%)	<0.001
Patients with Gd-Enhancing Lesions	44 (44.9%)	16 (16.3%)	<0.001

MRI—Magnetic Resonance Imaging.

**Table 8:** MRI findings at baseline and two years stratified by MS subtype.

MRI Variable	Timepoint	RRMS (n = 80), n (%)	SPMS (n = 18), n (%)	*p*-Value (Between Groups)
Brain Atrophy	Baseline	20 (25.0%)	11 (61.1%)	0.007
	Two Years	25 (31.3%)	18 (100.0%)	<0.001
Corpus Callosum Atrophy	Baseline	15 (18.8%)	6 (33.3%)	0.296
	Two Years	21 (26.3%)	13 (72.2%)	0.001
Black Holes	Baseline	19 (23.8%)	7 (38.9%)	0.308
	Two Years	23 (28.8%)	16 (88.9%)	<0.001
Cervical Confluent Lesions	Baseline	11 (13.8%)	5 (27.8%)	0.27
	Two Years	13 (16.3%)	12 (66.7%)	<0.001

RRMS—Relapsing-Remitting Multiple Sclerosis; SPMS—Secondary Progressive Multiple Sclerosis; MRI—Magnetic Resonance Imaging.

**Table 9:** Predictors of disease progression over two years.

Predictors	Odds Ratio	95% Confidence Interval	*p*-Value
Baseline Brain Atrophy (Yes vs. No)	2.6	1.25–5.40	0.012
Baseline EDSS Score	1.35	1.12–1.60	0.001
Age at Diagnosis	1.05	1.01–1.09	0.018
Previous Natalizumab Use (Yes vs. No)	0.58	0.32–0.88	0.038
Gender (Male vs. Female)	1.12	0.72–1.85	0.63
MS Subtype (SPMS vs. RRMS)	2.1	0.95–4.60	0.075

Disease progression was considered as confirmed disability progression; EDSS—Expanded Disability Status Scale; MS—Multiple Sclerosis; RRMS—Relapsing-Remitting Multiple Sclerosis; SPMS—Secondary Progressive Multiple Sclerosis.

## Data Availability

Data are available on request.
